# Selection of reference genes for quantitative real-time PCR in bovine preimplantation embryos

**DOI:** 10.1186/1471-213X-5-27

**Published:** 2005-12-03

**Authors:** Karen Goossens, Mario Van Poucke, Ann Van Soom, Jo Vandesompele, Alex Van Zeveren, Luc J Peelman

**Affiliations:** 1 Department of Animal Nutrition, Genetics, Breeding and Ethology, Faculty of Veterinary Medicine, Ghent University, Heidestraat 19, B-9820 Merelbeke, Belgium; 2 Department of Reproduction, Obstetrics and Herd Health, Faculty of Veterinary Medicine, Ghent University, Salisburylaan 133, B-9820 Merelbeke, Belgium; 3 Center for Medical Genetics Ghent, Ghent University Hospital, Medical Research Building, De Pintelaan 185, B-9000 Ghent, Belgium

## Abstract

**Background:**

Real-time quantitative PCR is a sensitive and very efficient technique to examine gene transcription patterns in preimplantation embryos, in order to gain information about embryo development and to optimize assisted reproductive technologies. Critical to the succesful application of real-time PCR is careful assay design, reaction optimization and validation to maximize sensitivity and accuracy. In most of the studies published *GAPD*, *ACTB *or *18S rRNA *have been used as a single reference gene without prior verification of their expression stability. Normalization of the data using unstable controls can result in erroneous conclusions, especially when only one reference gene is used.

**Results:**

In this study the transcription levels of 8 commonly used reference genes (*ACTB*, *GAPD*, *Histone H2A*, *TBP*, *HPRT1*, *SDHA*, *YWHAZ *and *18S rRNA*) were determined at different preimplantation stages (2-cell, 8-cell, blastocyst and hatched blastocyst) in order to select the most stable genes to normalize quantitative data within different preimplantation embryo stages.

**Conclusion:**

Using the geNorm application *YWHAZ*, *GAPD *and *SDHA *were found to be the most stable genes across the examined embryonic stages, while the commonly used *ACTB *was shown to be highly regulated. We recommend the use of the geometric mean of those 3 reference genes as an accurate normalization factor, which allows small expression differences to be reliably measured.

## Background

Preimplantation bovine embryo development is characterized by distinct biological steps, including first cleavage division, activation of the embryonic genome, compaction and blastocyst formation with the derivation of two different cell lines, the inner cell mass and the trophectodermal cells. These processes are regulated by differential expression of developmentally important genes, mostly expressed in a stage- and time-dependent manner following the common maternal and/or embryonic expression pattern [1]. The acquisition of knowledge about the physiological timetable of gene expression during preimplantation development is crucial for a better understanding of mammalian embryo development and is useful for further refinement of assisted reproductive technology in mammals [2].

Until recently, most studies of oocyte and embryo physiology were based on microscopic observations, but it is commonly agreed that the evaluation of embryo morphology alone does not answer most of the questions [3]. New insights into preimplantation development were gained through the measurement of differential mRNA levels in oocytes and preimplantation embryos by Reverse Transcription (RT-) PCR methods [4-7] that replace less sensitive and more laborious methods like Northern blot analysis and RNase protection assay. However due to differential reaction efficiencies and kinetics in RT-PCR, the amount of final product after amplification may not accurately reflect the initial sample mRNA concentration [8].

Real-time RT-PCR assays in which data are accurately normalized, are significantly less variable than commonly used conventional RT-PCR procedures. Real-time quantification at the exponential phase is not affected by any reaction components becomming limited in the plateau phase. Although small differences in transcript levels can be measured by endpoint RT-PCR, a lot of optimizations and post-PCR manipulations are required. These optimizations have to be performed for every individual sample, because the RNA expression can vary a lot between individual samples. Therefore, real-time RT-PCR has been recognised as the method of choice for accurate and sensitive quantification of mRNA transcripts [9,10]. Many studies have now been published where RNA quantification has been assessed in early domestic animal embryos. This technique has the advantage of speed, high throughput and accuracy over a large dynamic range of quantification and is especially suitable when only a small number of cells are available [11]. Several authors [12-14] have demonstrated the reproducibility of the 2 step SYBR Green I real-time RT-PCR reaction by determination of the intra- and interassay variation.

However, a lack of standards, variation in assay design, diversity of protocols, instruments and analysis methods make that real-time qRT-PCR results should be treated with caution and that agreed standards and operating procedures are required [15].

Several variables need to be controlled for gene-transcription analysis, such as the amount of starting material, enzymatic efficiencies, and differences between tissues or cells in overall transcriptional activity. Many methods are used to control some of these these variables, for example normalization against the total cell number, against the mass of the input material or against the RNA mass quantity. Exogenously added mRNA or spikes can be used for standardization when the spike is added before the RNA extraction [16].

Normalization against internal control genes is most frequently used because it can control all variables. Those internal control genes, also known as reference genes, are often referred to as housekeeping genes, assuming that those genes are expressed at a constant level in certain tissues, at all stages of development and are unaffected by the experimental treatment. To date most of the standardizations are done to reference genes such as β-actin (*ACTB*), glyceraldehyde 3-phosphate dehydrogenase (*GAPD*) or *18S rRNA*. However a number of studies have provided solid evidence that their transcription levels are not constant between different developmental stages and different experimental conditions [17-19]. Normalization of the data using these types of apparent controls can result in false conclusions being made regarding transcription levels. Therefore, validation of candidate reference genes is critical for accurate analysis of gene expression [20].

Most experiments include only a single reference gene. Vandesompele *et al*. [21] demonstrated that the conventional use of a single gene for normalization leads to relative large errors and they validated the geometric mean of multiple carefully selected reference genes as an accurate normalization factor.

In this study, we determined the mRNA expression levels of 8 commonly used reference genes at different preimplantation stages and calculated a normalization factor based on multiple control genes for more accurate and reliable normalization of gene-expression data in bovine preimplantation embryos.

## Results

### Sample quality

For each assay, embryos with good morphological characteristics [22] were selected from 3 different in vitro embryo production (IVP) experiments. The mean percentage of obtained embryos from all cultivated oocytes at the different developmental stages were 65 ± 6% for the 2-cell stage, 44 ± 5% for the 8-cell stage, 25 ± 4% for the blastocyst stage and 16 ± 3% for the hatched blastocyst stage. Because IVP is time consuming and only a restricted amount of fertilized oocytes develop to the desired embryonic stages, the number of assays was restricted to 3.

Total RNA was isolated from pools of 20 embryos per assay and for each examined developmental stage. Because of the very small cell numbers used for RNA extraction, the RNA quantity could not be measured by the BioPhotometer (Eppendorf, Leuven) or the Nanodrop ND 1000 spectrophotometer.

A minus RT control demonstrated the presence of a considerable amount of contaminating genomic DNA. This illustrated the necessity of a DNase treatment, which removed all the contaminating genomic DNA from the RNA samples.

Although the RNA quality and quantity could not be determined, a real-time PCR with the reference gene *GAPD *gave cycle threshold (Ct) values in the range of 23 to 27 and a single band on agarose gel. This first-strand cDNA was 2.5 times diluted with 10 mM Tris HCl pH 8 and used for further real-time applications.

### Transcription profiling of the reference genes

An initial screening of the transcription profiles of the selected reference genes by RT-PCR showed that all of those genes were expressed across the preimplantation embryo stages of interest. None of the eight selected genes was excluded from the study.

Gene-specific amplification was confirmed by a single peak in melt-curve analysis and a single band with the expected size in agarose gel electrophoresis. No primer-dimer formation was detected and the identities of the PCR products were confirmed by sequencing (Table 1).

**Table 1:** Information on the primers used for real-time PCR

Gene	Genbank Accession number	species	Sequence	Product size (bp)	Ta (°C)	% homology
*ACTB*^[23]^	AY141970	Cow	5'-CCTCACGGAACGTGGTTACA-3' 5'-TCCTTGATGTCACGCACAATTT-3'	87	58	100 %
*GAPD*	XM_618013	Cow	5'-TTCAACGGCACAGTCAAGG-3' 5'-ACATACTCAGCACCAGCATCAC-3'	119	62	100 %
*Histone H2A*^[15]^	U62674	Mouse	5'-GTCGTGGCAAGCAAGGAG-3' 5'-GATCTCGGCCGTTAGGTACTC-3'	182	60	82 %
*TBP*^[33]^	NM_003194	Human	5'-CCTAAAGACCATTGCACTTCG-3' 5'-CTTCACTCTTGGCTCCTGTG-3'	146	57	94 %
*HPRT1*	AF176419	Cow	5'-TGCTGAGGATTTGGAGAAGG-3' 5'-CAACAGGTCGGCAAAGAACT-3'	154	58	100 %
*SDHA*	NM_174178	Cow	5'-GCAGAACCTGATGCTTTGTG-3' 5'-CGTAGGAGAGCGTGTGCTT-3'	185	60	100 %
*YWHAZ*	BM446307	Cow	5'-GCATCCCACAGACTATTTCC-3' 5'-GCAAAGACAATGACAGACCA-3'	120	60	97 %
*18S rRNA*	AF176811	Cow	5'-AGAAACGGCTACCACATCCA-3' 5'-CACCAGACTTGCCCTCCA-3'	169	62	100 %

For all genes studied, standard curves derived from 10-fold serial dilutions of pooled cDNA gave correlation coefficients greater than 0.97 and efficiencies greater than 90%. The reaction efficiencies were used to transform the Ct-values into raw data for analysis with the geNorm software [21].

Three identical real-time qPCR assays were performed. In each assay the transcription levels of the selected reference genes were measured in duplicate, at 4 different stages of preimplantation development.

To compare the RNA transcription levels across the stages of embryonic development, the Ct values were compared. The Ct-value is defined as the number of cycles needed for the fluorescence signal to reach a specific threshold level of detection and is inversely correlated with the amount of template nucleic acid present in the reaction [23]. Most of the genes had Ct values in the range of 25 to 33 but *18S rRNA *was more abundant (Ct levels <15).

### GeNorm analysis

Analysis of the gene expression stability over the different embryonic stages was done using the geNorm software. The ranking of the 8 control genes according to their M value was equivalent between the 3 assays. *GAPD*, *YWHAZ*, *SDHA *and *18S rRNA *were the 4 most stable genes in each of the 3 assays, only the order of the genes was different. *ACTB *was the least stable gene in the 3 assays. The results are listed in Table 2.

**Table 2:** Ranking of the reference genes in order of their expression stability per assay, decreasing from top to bottom. The reference genes chosen to calculate the normalization factor are printed in bold.

Assay 1	Assay 2	Assay 3	Combined*
***SDHA***	***SDHA***	***GAPD***	***GAPD***
*18S rRNA*	***GAPD***	***YWHAZ***	***YWHAZ***
***GAPD***	*18S rRNA*	*18S rRNA*	*18S rRNA*
***YWHAZ***	***YWHAZ***	***SDHA***	***SDHA***
*HPRT1*	*Histone H2A*	*Histone H2A*	*Histone H2A*
*Histone H2A*	*HPRT1*	*TBP*	*HPRT1*
*TBP*	*TBP*	*HPRT1*	*TBP*
*ACTB*	*ACTB*	*ACTB*	*ACTB*

*After correction for run-to-run variation

**Figure 1:**
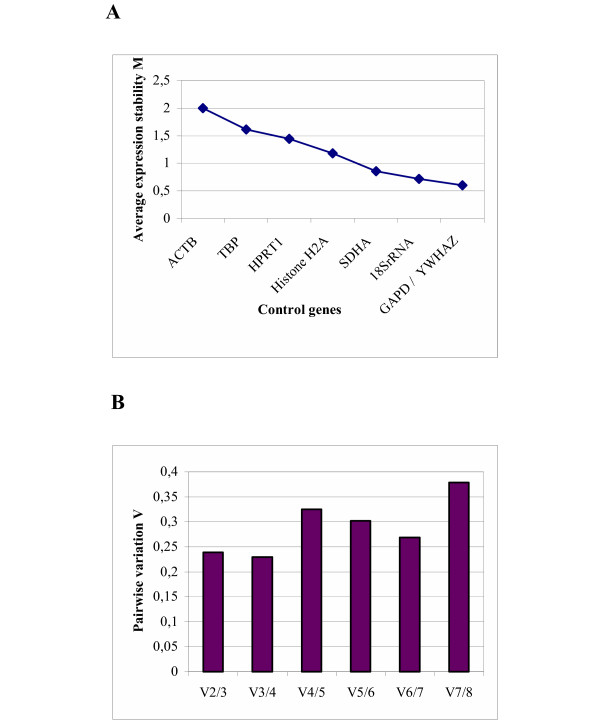
**(A-B): Gene expression stability of the candidate reference genes analyzed by the geNorm program**. (A) Average expression stability values (M) of the control genes over the 3 assays together, plotted from least stable (left) to most stable (right). (B) Pairwise variation analysis over the 3 assays together between the normalization factors NF_n _and NF_n+1_, to determine the optimal number of control genes for normalization. The higher V_4/5 _and V_7/8 _values are due to the inclusion of a relative unstable gene and are in accordance with the average expression stability M.

To ensure comparability between the 3 assays, we compared the Ct values and efficiencies of the relative standard curves, derived from the same pooled cDNA stock, between the three independent assays and made a correction for the plate-to-plate variation according to the qBase algorithm (Hellemans *et al*., in preparation) [24]. This correction factor for inter-assay variation was necessary for the determination of the most stable reference gene over the 3 assays together. The results of the geNorm analysis of the combined assay are shown in Figure 1A. The ranking of the genes in this combined assay is in agreement with the ranking of the 3 individual assays.

To determine how many reference genes should be used, normalization factors (NF_n_), based on the geometric mean of the expression levels of the n best reference genes, were calculated by stepwise inclusion of an extra, less stable reference gene according to Vandesompele *et al*. [21]. Figure 1B shows the pairwise variation V_n_/V_n+1 _between 2 sequential normalization factors NF_n _and NF_n+1_. A large variation means that the added gene has a significant effect and should probably be included for calculation of the normalization factor. In this case, the inclusion of a 4^th ^gene has no significanf effect (low V_3/4 _value) on the NF. The 3 member set *GAPD*, *SDHA *and *YWHAZ *is an excellent choice for the calculation of the NF.

## Discussion

Analysis of expression patterns of genes essential in early embryo development, provides a useful tool to assess the normality of the embryos and a tool to optimize assisted reproduction technologies [25]. New insights into early embryo development of mammals are commonly gained through the measurement of different mRNA levels by real-time qPCR. This technique has revolutionized the quantification of mRNA but requires careful assay design and reaction optimization to maximize sensitivity, accuracy and precision [26].

The problem of measuring transcript levels throughout preimplantation development is confounded by the fact that cell numbers and cell sizes are constantly changing during this developmental interval. Untill the maternal-zygotic transition, the mRNA is mainly of maternal origin. Once the genome is activated, the cell number will influence the amount of mRNA available [3]. To allow ontogenic analysis, the embryos were compared as a single unit and the reference genes will correct for the differences between the embryos.

Using in vitro culture, another variable that must be taken into account is the proportion of normal embryos in the sample. Incompetent embryos may be mixed with competent embryos during the analysis [3]. The gene expression in incompetent embryos may be different from those in competent embryos and could introduce a bias [27-29]. As such, there might be an influence of abnormal embryos on the choice of reference genes when using single embryo samples. Therefore we used groups of 20 pooled embryos with good morphological characteristics to minimize the influence of the quality of the individual embryo.

RNA quality and quantity are critical for succesful gene expression analysis. Due to the limited amount, RNA analysis was not possible, but an RNA extraction method optimized for small sample quantities, DNase treatment and appropriate control methods resulted in as reliable as possible results.

Critical to the successful application of real-time qPCR is the prevention of amplification of contaminating genomic DNA, resulting in an overestimation of the amount of RNA present. But even more importantly, yielding unreliable data especially for low abundant single exon genes or genes with retropseudogenes in the genome. Minus RT-controls before and after the DNAse treatment demonstrated the necessity and efficacy of the DNase treatment.

When intercalating dyes such as SYBR green I are used, attention should be paid to the formation of primer-dimers. Melting curve analysis and agarose gel electrophoresis confirmed that the fluorescent signal was specifically from the desired amplicons, not from artefacts.

Accurate normalization is required to correct real-time data for differences in cellular input, RNA quality and enzymatic efficiency between the samples. Under controlled conditions of reproducable extraction of good-quality RNA, the gene transcript number is ideally standardized to the number of cells [21]. During the bovine preimplantation period the cell numbers and cell size are constantly changing. Comparing mRNA levels within the same developmental stage is feasible but doing ontogenic analyses, which are essential to understand transitions in gene expression, is more problematic [3].

Another way of normalization is using the mass of the input material. However, in our case it was impossible to quantify those parameters because only minimal amounts of RNA were available and the total amount of RNA present throughout the preimplantation period is not constant. So normalization to total RNA requires a reliable RNA quantification method, and fails to take into account the variability of the RT-reaction. Probably the strongest argument against the use of total RNA mass for normalization is the fact that it predominantly consists of rRNA molecules and is not always representative of the mRNA fraction [21].

The addition of exogenously added mRNA can only be used when the exact amount of cells or starting material is known [16]. Spikes only correct for differences in enzymatic efficiencies but do not account for the quality and quantity of the input sample. Therefore, the use of spikes is not assumption free.

It is now generally accepted that transcription levels should be normalized to an invariable internal control gene. Those reference genes are often adapted from the literature and used against a variety of experimental conditions. An ideal reference gene should be expressed at a constant level among different experimental conditions and at all stages of development. An ontogenic study of several commonly used reference genes showed that their mRNA levels are not stable throughout preimplantation development [19]. If the used reference gene fluctuates between the samples, the subsequent normalization will cause erroneous results [30]. As the biological function of many genes is still unknown, it is difficult to predict how experimental conditions will affect the expression of the putative reference genes. Thus a safer approach is to use the geometric average expression of several genes that show small variance. Vandesompele *et al*. [21] postulated that gene pairs that have stable expression patterns relative to each other are proper control genes. *YWHAZ*, *GAPD*, *SDHA *and *18S rRNA *were found to be the best endogenous control genes in preimplantation embryo samples as represented by their low M values, the marker of gene stability. *ACTB *was the worst scoring reference gene, in the set of 8 tested reference genes (Figure 1A). This is a remarkable result given the fact that in several publications on gene expression analysis in embryos, *ACTB *was the only reference gene used. However, the differential mRNA expression of *ACTB *is in accordance with previous reports that prove the upregulation of *ACTB *during preimplantation embryo development and predict a role for *ACTB *during blastocyst formation [31-33]. In previous studies *Histone H2A *was determined as the most stable reference gene during preimplantation embryo development [8,19], but those authors only considered Ct values and did not correct for the amount of input material. To validate the presumed stable expression of a given control gene, prior knowledge of a reliable measure to normalize this gene in order to remove any nonspecific variation is required. To address this circular problem, Vandesompele *et al*. [21] developed a gene-stability measure to determine the expression stability of control genes on the basis of non-normalized expression levels. By using this approach, the changing RNA content during the developmental stages was taken into account [34]. Besides geNorm, other programs and strategies are described in literature to select the best reference genes. BestKeeper [35] is an Excel-application also based on pairwise correlation. Normfinder [13] is a model based approach and enables estimation not only of the overall variation of the candidate normalization genes, but also of the variation between sample subgroups of the sample set.

A normalization factor (NF) based on the geometric mean of the best performing reference genes was calculated. The number of genes used to calculate this NF is a trade-off between practical considerations and accuracy. In this case, the 3 most stable reference genes were used to calculate the normalization factor (NF_3_). Figure 1B demonstrates that the inclusion of a 4^th ^reference gene has no significant contribution to the newly calculated normalization factor NF_4_.

Ultimately, our choice for the normalizing set is the geometric mean of the transcription levels of *GAPD*, *SDHA *and *YWHAZ*. *18S rRNA *was excluded because rRNA genes have general disadvantages when used as reference genes. Their transcription is carried out by RNA polymerase I, therefore the regulation of rRNA synthesis is independent from mRNA synthesis, which is carried out by RNA polymerase II [36]. Besides, rRNA genes are highly abundant compared to the target mRNA transcripts, this imbalance makes it difficult to accurately subtract the baseline values in real-time qPCR analysis [21]. *18S rRNA *was evaluated in this study because it is a commonly used reference gene.

## Conclusion

In conclusion, a method for gDNA free RNA extraction from embryos was optimized and a reference gene assay for reliable normalization of real-time PCR data, obtained from bovine preimplantation embryo samples was designed. Transcription profiling of 8 different reference genes showed that the use of a single reference gene is not reliable and will result in erroneous conclusions. Instead *GAPD*, *SDHA *and *YWHAZ *should be used.

## Methods

### In vitro production of bovine embryos

Bovine embryos were produced by routine in vitro methods as described by Yuan and colleagues [37]. Briefly, bovine oocytes were obtained from ovaries collected at a local slaughterhouse. Immature cumulus-oocyte complexes were selected from follicular fluid, washed three times in HEPES-TALP and matured for 22 to 26 h in groups of 100 in 500 μl maturation medium at 39°C in a humified 5% CO_2 _incubator. After maturation the oocytes were inseminated with frozen-thawed sperm of a dairy bull (1 × 10^6 ^spermatozoa/ml). The cumulus cells and spermatozoa were mechanically removed from the presumptive zygotes, which were placed in groups of 25 in 50 μl droplets of synthetic oviduct fluid supplemented with 5% fetal calf serum and cultured up to the desired stages. The embryos were collected at the indicative time period after fertilization: 2-cell (24–36 h), 8-cell (48–64 h), blastocyst (day 7) and hatched blastocyst (day 8). All embryos were washed three times in PBS, collected in pools of 20 and frozen at -80°C until RNA extraction.

### RNA extraction and cDNA synthesis

Total RNA was isolated from 20 pooled embryos using the PicoPure RNA Isolation Kit (Arcturus, Mountain View, CA) according to the manufacturer's instructions. This kit is engineered to recover high-quality total RNA from pico-scale samples.

For genomic DNA removal an in-solution DNase digestion was carried out by treating the total RNA with 2 units of RQ1 DNase (Promega, Leiden) followed by a spin-column purification (Microcon YM-100, Millipore, Brussels). A minus RT control was performed with primers for *GAPD *to check the removal of all the contaminating genomic DNA.

First-strand cDNA was synthesized from the total amount of RNA using the iScript cDNA synthesis kit (Bio-Rad, Nazareth), following the manufacturer's instructions. The iScript Reverse Transcriptase is a modified MMLV-derived reverse transcriptase and the iScript Reaction Mix contains both oligo(dT) and random primers. After the RT reaction and RT control with primers for *GAPD*, the cDNA was 2.5 times diluted in 10 mM Tris HCl pH 8.0.

### Reference gene selection and primer design

Eight reference genes were selected (*ACTB*, *GAPD*, *Histone H2A*, *TBP*, *HPRT1*, *SDHA*, *YWHAZ *and *18S rRNA*) that belong to different functional classes to reduce the chance that the genes might be co-regulated (Table 3).

**Table 3:** Functions of the selected reference genes

Symbol	Gene name	Function
*ACTB*	β-actin	Cytoskeletal structural protein
*GAPD*	Glyceraldehyde-3-phosphate dehydrogenase	Glycolytic enzyme
*Histone H2A*	Histone 2 alpha	Nucleosome structure
*TBP*	TATA box binding protein	General RNA polymerase II transcription factor
*HPRT1*	Hypoxanthine phosphoribosyl-transferase I	Purine synthesis in salvage pathway
*SDHA*	Succinate dehydrogenase flavoprotein subunit A	Electron transporter in the TCA cycle and respiratory chain
*YWHAZ*	Tyrosine 3-monooxygenase/tryptophan 5-monooxygenase activation protein, zeta polypeptide	Signal transduction by binding to phosphoserine-containing proteins
*18S rRNA*	18S ribosomal RNA	Ribosome unit

Primers for *Histone H2A *were taken from Robert and colleagues [19], primers for *TBP *were taken from Vigneault and colleagues [38] and primers for *ACTB *were taken from Fair and colleagues [27]. The other primers were designed by the Primer 3 software [39] and were based on RNA or DNA sequences found in Genbank. The reported bovine sequences were preferentially used and the specificity of the primers was tested using a BLAST analysis against the genomic NCBI database. PCR amplicons were characterized using Mfold [40] in order to predict the nature of any secondary structures which might influence the PCR efficiency. The PCR products were cloned (pCR 2.1 vector, Invitrogen, Merelbeke) and sequenced for verification (Thermo Sequenase Primer Cycle Sequencing Kit, Amersham Bioscience, Roosendaal) with a ALF Express sequencer (Amersham Bioscience, Roosendaal) [GenBank: DQ066891, DQ066892, DQ066893, DQ066894, DQ066895, DQ066896, DQ066897 and DQ066898]. Primer and amplicon information are listed in Table 1.

### Real-Time quantitative PCR

Three replicates of 20 pooled embryos were used for each developmental stage (2-cell, 8-cell, blastocyst and hatched blastocyst) as described by Robert et al. [19].

All PCR reactions were performed in a 15 μl reaction volume on the iCycler iQ Real-Time PCR Detection System (Bio-Rad, Nazareth) using the iQ SYBR Green Supermix (Bio-Rad, Nazareth), 200 nM of each specific primer and 2.5 μl of diluted cDNA or one embryo equivalent per reaction.

The PCR program consisted of an initial denaturation step at 95°C for 3 minutes to activate the *Taq *DNA polymerase, followed by 45 cycles of denaturation at 95°C for 20 seconds and a combined primer annealing/extension at the specific annealing temperature for 40 seconds during which fluorescence was measured. A melt curve was produced to confirm a single gene-specific peak and to detect primer/dimer formation by heating the samples from 70 to 95°C in 0.5°C increments with a dwell time at each temperature of 10 seconds while continuously monitoring the fluorescence. PCR efficiencies were calculated using a relative standard curve derived from a pooled cDNA mixture (a ten-fold dilution series with four measuring points). This pooled cDNA was obtained from bovine heart, kidney, liver, muscle, lung and placenta tissue, using Total RNA Isolation Reagent (TRIR, ABgene, Epsom) for the RNA isolation and the iScript cDNA synthesis kit (Bio-Rad, Nazareth) for the RT-reaction.

Each reaction was run in duplicate, whereby a no-template control was included.

### Determination of reference gene expression stability

To determine the stability of the selected reference genes, the geNorm Visual Basic application for Microsoft Excel was used as described by Vandesompele *et al*. [21].

This approach relies on the principle that the expression ratio of two perfect reference genes should be identical in all samples, regardless of the experimental condition or cell type. Increasing variation in this ratio corresponds to decreasing expression stability. The program calculates the gene stability measure M by determining the average pair-wise variation between a particular reference gene and all other control genes. Genes with higher M values have greater variation in RNA expression. By stepwise exclusion of the least stable gene and recalculation of the M values, the most stable reference genes are identified. Finally, a normalisation factor (NF) was calculated based on the geometric mean of the expression levels of the best-performing reference genes.

## Authors' contributions

KG performed all the experimental procedures and was the primary author of the manuscript. MVP participated in the study design and provided real-time support. AVS contributed to the IVF experiments. JV provided expert input in data analysis. AVZ and LJP participated in the design of the project, helped to draft the manuscript and supervised the study. All authors read and approved the final manuscript.
